# Retraction: Association Between Baseline Aortic 18F-Fluorodeoxyglucose Uptake and Subsequent Aortic Dilatation in Giant Cell Arteritis: A Four-Year Post Hoc Analysis

**DOI:** 10.7759/cureus.r247

**Published:** 2026-09-18

**Authors:** Michelle Villarreal Compagny

**Affiliations:** 1 Internal Medicine, Hospital Clínic de Barcelona, Barcelona, ESP

This article has been retracted at the request of the author and with the agreement of the Editor-in-Chief. The article reports an analysis of institutional cohort data for which authorization for use could not be documented.

